# The effect of iodine supplementation in pregnancy on early childhood neurodevelopment and clinical outcomes: results of an aborted randomised placebo-controlled trial

**DOI:** 10.1186/s13063-015-1080-8

**Published:** 2015-12-10

**Authors:** Shao J. Zhou, Sheila A. Skeaff, Philip Ryan, Lex W. Doyle, Peter J. Anderson, Louise Kornman, Andrew J. Mcphee, Lisa N. Yelland, Maria Makrides

**Affiliations:** Women’s & Children’s Health Research Institute, 72 King William Road, North Adelaide, SA 5006 Australia; School of Agriculture, Food & Wine, University of Adelaide, Waite Campus, Waite Road, Urrbrae, SA 5064 Australia; Department of Human Nutrition, University of Otago, PO Box 56, Dunedin, 9054 New Zealand; School of Population Health, University of Adelaide, 178 North Terrace, Adelaide, SA 5005 Australia; Department of Obstetrics and Gynaecology, Royal Women’s Hospital, Cnr of Flemington Road and Grattan Street, University of Melbourne, Melbourne, VIC 3052 Australia; Clinical Sciences, Murdoch Childrens Research Institute, Flemington Road, Melbourne, VIC 3052 Australia; Department of Paediatrics, University of Melbourne, 50 Flemington Road, Melbourne, VIC 3052 Australia; Women’s and Children’s Health Network, 72 King William Road, North, Adelaide, SA 5006 Australia; School of Paediatrics & Reproductive Health, University of Adelaide, Frome Road, Adelaide, SA 5005 Australia; South Australian Health and Medical Research Institute, North Terrace, Adelaide, SA 5000 Australia

**Keywords:** Iodine, Supplementation, Pregnancy, Child development, RCT

## Abstract

**Background:**

Concern that mild iodine deficiency in pregnancy may adversely affect neurodevelopment of offspring has led to recommendations for iodine supplementation in the absence of evidence from randomised controlled trials. The primary objective of the study was to investigate the effect of iodine supplementation during pregnancy on childhood neurodevelopment. Secondary outcomes included pregnancy outcomes, maternal thyroid function and general health.

**Methods:**

Women with a singleton pregnancy of fewer than 20 weeks were randomly assigned to iodine (150 μg/d) or placebo from trial entry to birth. Childhood neurodevelopment was assessed at 18 months by using Bayley Scales of Infant and Toddler Development (Bayley-III). Iodine status and thyroid function were assessed at baseline and at 36 weeks’ gestation. Pregnancy outcomes were collected from medical records.

**Results:**

The trial was stopped after 59 women were randomly assigned following withdrawal of support by the funding body. There were no differences in childhood neurodevelopmental scores between the iodine treated and placebo groups. The mean cognitive, language and motor scores on the Bayley-III (iodine versus placebo, respectively) were 99.4 ± 12.2 versus 101.7 ± 8.2 (mean difference (MD) −2.3, 95 % confidence interval (CI) −7.8, 3.2; *P* = 0.42), 97.2 ± 12.2 versus 97.9 ± 11.5 (MD −0.7, 95 % CI −7.0, 5.6; *P* = 0.83) and 93.9 ± 10.8 versus 92.4 ± 9.7 (MD 1.4, 95 % CI −4.0, 6.9; *P* = 0.61), respectively. No differences were identified between groups in any secondary outcomes.

**Conclusions:**

Iodine supplementation in pregnancy did not result in better childhood neurodevelopment in this small trial. Adequately powered randomised controlled trials are needed to provide conclusive evidence regarding the effect of iodine supplementation in pregnancy.

**Trials registration:**

The trial was registered with the Australian New Zealand Clinical Trials Registry at http://www.anzctr.org.au. The registration number of this trial is ACTRN12610000411044. The trial was registered on 21 May 2010.

## Background

Iodine is essential for the production of thyroid hormones. Iodine deficiency encompasses a spectrum of disorders, including impaired growth and neurodevelopment [1]. Pregnant women have a higher risk of iodine deficiency because of their increased iodine requirement [2]. Severe iodine deficiency during pregnancy causes cretinism and irreversible brain damage in the offspring [1]. There is increasing concern that mild to moderate iodine deficiency during pregnancy—which has emerged as a public health issue in a number of developed countries, including Australia and the UK—may lead to cognitive deficits and learning disability in children.

A recent systematic review of randomised controlled trials (RCTs) highlighted that the effect of iodine supplementation in pregnancy in regions with mild to moderate iodine deficiency is unclear because none of the RCTs conducted in those regions assessed developmental outcomes of children [3]. There is some evidence from non-randomised intervention studies suggesting that iodine supplementation in pregnancy in regions of mild to moderate iodine deficiency may improve cognitive function in children [4]. Conversely, adverse effects on child development in relation to iodine supplementation in pregnancy have also been reported from cohort studies [5]. These emerging data have been differentially interpreted by expert groups and government authorities worldwide, resulting in various approaches to address this public health issue.

The American Thyroid Association and the European Thyroid Association have recommended routine iodine supplementation in pregnancy [6, 7], whereas the recommendation for iodine supplements in pregnancy by the World Health Organization (WHO) is dependent on the iodised salt coverage and the iodine status of the population [8]. There are no specific recommendations from the government authorities in the UK or USA. In Australia and New Zealand, mandatory iodine fortification of bread was implemented in 2009. In addition, the Australian National Health and Medical Research Council (NHMRC) recommended that all pregnant women take an iodine supplement of 150 μg/d [9] because of concerns that the mandatory iodine fortification may not be adequate to prevent iodine deficiency in pregnant women [10]. The present study was designed as a double-blind placebo-controlled multi-centre RCT in Australia and New Zealand. The aim of the study was to assess the effect of iodine supplementation in pregnancy over and above the mandatory iodine fortification on childhood neurodevelopment and other clinical outcomes, including pregnancy outcomes, maternal thyroid function, mental health and general well-being.

## Methods

### Participants and recruitment

Pregnant women were approached to enter the trial at their first antenatal visit. They were eligible if they were less than 20 weeks’ gestation with a singleton pregnancy. Women were excluded if they were taking a supplement containing iodine, had a history of thyroid disease or drug or alcohol abuse, their fetus had a known major abnormality, or if English was not the main language spoken at home. Ethical approval was obtained from the Human Research Ethics Committee at each participating centre (Children, Youth & Women’s Health Service Research Ethics Committee and the Flinders Clinical Research Ethics Committee), and written informed consent was obtained from each participant. The trial was registered on the Australian and New Zealand Clinical Trials Registry (#ACTRN12610000411044).

### Randomisation and treatment

Women were randomly assigned to iodine or placebo in the ratio of 1:1 through a web-based randomisation service. The randomisation schedule was generated independently with balanced, variable-sized blocks and was stratified by centre, parity (0 versus ≥1), and gestational age at randomisation (≤16 weeks’ versus >16 weeks’). Neither the women nor the research staff were aware of the women’s group allocation. The iodine supplements contained 150 μg of iodine per tablet as potassium iodide, whereas the placebo tablets contained no iodine. Women were asked to take one trial tablet daily from randomisation to the end of their pregnancy. The trial tablets were manufactured and donated by Blackmores (Warriewood, Australia). All tablets were similar in size, shape, smell and colour. Women were supplied with excess tablets and were asked to return any unused tablets at the end of the pregnancy as a measure of compliance. Regular telephone calls during the intervention period were made to monitor adverse side effects and encourage compliance.

### Outcome assessments

#### Childhood neurodevelopment

The primary outcome of childhood neurodevelopment was assessed at 18 months of age by using the cognitive, language and motor composites of the Bayley Scales of Infant and Toddler Development, third edition (Bayley-III) [11]. Composite scores are age-standardised with a normative mean of 100 and a standard deviation of 15. The standardised scores were also classified into the categories of any developmental delay (<85) and moderate/severe developmental delay (<70)*.* The social-emotional behaviours and adaptive behaviours scales of the Bayley-III were also administered. Bayley-III was used to assess childhood development as it is the most widely used objective measure of early development. It is used extensively in research including neonatal trials and has a moderate association with later intelligence quotient (IQ) [12].

#### Iodine status and thyroid function

At study entry and 36 weeks’ gestation, women were asked to collect a spot urine sample to assess urinary iodine concentration (UIC). UIC was determined by using the modified WHO Method A [13]. A blood sample was also collected at baseline by venepuncture to assess thyroid hormone concentrations, including thyroid-stimulating hormone (TSH), thyroglobulin (Tg), free triiodothyronine (fT3) and free thyroxine (fT4). Thyroid function of newborns was also assessed from cord blood (TSH, fT3, fT4 and Tg) and from newborn screening (TSH only). A breast milk sample 6 weeks after birth was collected, where possible, to assess breast milk iodine concentration by inductively coupled plasma mass spectrometry method [14].

### Pregnancy and other clinical outcomes

Pregnancy and birth outcome data were collected by blinded review of medical records. Small and large for gestational age were defined as birth weight below the 10th and above the 90th percentile, respectively, for gestational age and sex [15]. Preterm birth was defined as gestational age at birth of fewer than 37 completed weeks. Gestational age was estimated on the basis of a composite of the last menstrual period and a dating ultrasound early in pregnancy, where available.

General health and well-being of women were assessed by using validated questionnaires, including the 36-Item Short Form Health Survey (SF-36) [16] and the Depression Anxiety Stress Scale (DASS) [17] at study entry, 36 weeks’ gestation and 6 weeks’ post-partum.

### Other assessments

Demographic characteristics were recorded at study entry. Safety of the intervention was assessed via telephone calls to women at 2 weeks after randomisation, and at 20, 28 and 36 weeks’ gestation, to assess potential side effects, including the frequency of sweating and palpitation, gastrointestinal side effects, including nausea, diarrhoea and constipation, as well as any serious adverse events defined as death or intensive care admission of either mother or baby.

### Sample size and statistical analysis

A sample size of 542 women per group was required to detect a minimum clinically meaningful difference in the Bayley-III composites of 4 points between the treatment groups with 90 % power, using a Bonferroni adjusted α = 0.017 for each of the three primary Bayley-III composites and allowing for adjustment for potential confounders and loss to follow-up. A 4-point difference was considered important in the context of other nutritional deficiencies and environmental exposures that have resulted in major public health campaigns [18, 19].

The primary analysis was based on the intention-to-treat principle by comparing the outcomes between the randomised treatment groups. Continuous outcomes were analysed by using *t* tests, or Kruskal-Wallis tests for non-normally distributed outcomes. Binary outcomes were analysed by using chi-squared tests, or Fisher’s exact tests for rare outcomes. Owing to a substantial sex imbalance between groups, a post hoc analysis was performed with adjustment for infant sex for the Bayley-III composites and anthropometric measurements at birth. No adjusted analysis was performed for other outcomes, because of the small sample size.

## Results

**Fig. 1:**
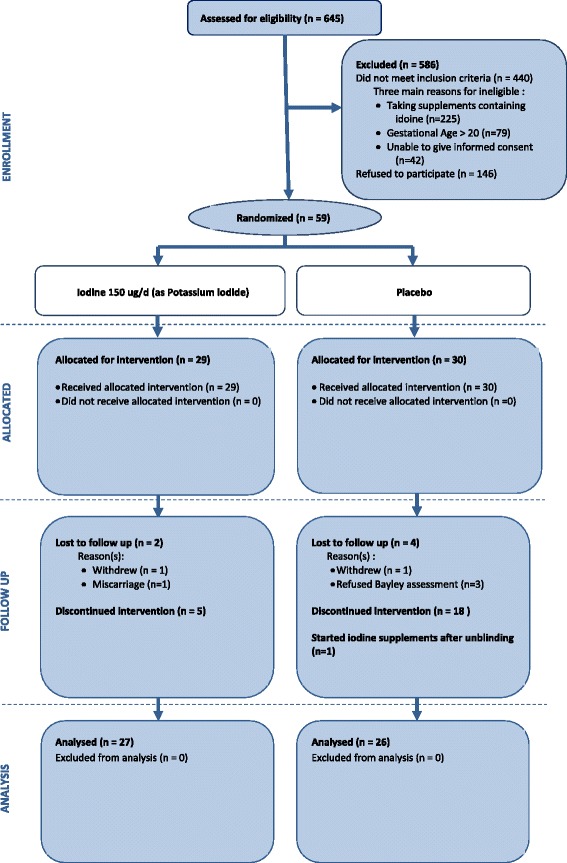
Participant flowchart

Of the 645 women approached, 205 met the eligibility criteria. A total of 59 out of 205 (29 %) women were enrolled in the study from the Women’s & Children’s Hospital and the Flinders Medical Centre in Adelaide, Australia, between June 2010 and October 2010 (Fig. 1). Twenty-nine were randomly allocated to iodine and 30 to placebo (Fig. 1). The baseline demographic characteristics of the participants are listed in Table 1. The trial was stopped early, before recruitment began in other Australian centres and New Zealand, because the funding body (NHMRC) withdrew its support for the trial. The NHMRC considered a placebo-controlled trial inconsistent with its recommendation for iodine supplementation in pregnancy. The ethics committees did not withdraw approval for the trial, but in view of the funding body’s position it supported the trial management committee’s decision to unblind the study and to follow all randomly assigned women as planned to monitor safety. Women were informed of their treatment group allocation and were provided with a copy of the NHMRC recommendation for iodine supplementation in pregnancy [9]. All women except two (one from each group) consented to continue with the follow-up after unblinding. The mean gestational age at unblinding was 33 ± 7 weeks. The mean duration of intervention before unblinding was 16 weeks (range 2–23 weeks). Nine (31 %) women in the iodine group and 4 (13 %) in the placebo group gave birth before unblinding. The decision regarding whether to continue taking trial supplements or to take commercially available iodine supplements was at the women’s discretion. Five women in the iodine group and 18 in the placebo group stopped taking trial supplements. Only one woman in the placebo group commenced iodine supplements after unblinding.

**Table 1:** Baseline demographic characteristics

	Iodine (*n* = 29)	Placebo (*n* = 30)
Age at trial entry, years^a^	29.1 (5.7)	29.8 (5.1)
Gestational age at trial entry, weeks^a^	15.2 (2.6)	14.9 (2.4)
Primiparous^b^	13 (44.8)	13 (43.3)
Completed secondary education^b^	20 (69.0)	22 (73.3)
Completed further education^b^	22 (75.9)	24 (80.0)
Smoke at trial entry or leading up to pregnancy^b^	7 (24.1)	5 (16.7)
Miscarriage in previous pregnancy^b^	8 (27.6)	12 (40.0)
Previous or current depression^b^	4 (13.8)	7 (23.3)
BMI at trial entry^a^	25.3 (5.9)	23.6 (3.9)
Pre-pregnancy BMI^a^	23.6 (5.5)	22.2 (4.3)
Infant sex: male^b^	20 (71.4)	9 (31.0)

*BMI* body mass index

^a^Data are presented as mean (standard deviation)

^b^Data are presented as number (percentage)

### Neurodevelopment of children

The mean composite score of the children did not differ between the iodine and the placebo groups in cognitive (99.4 ± 12.2 versus 101.7 ± 8.2, mean difference (MD): −2.3; 95 % confidence interval (CI) −7.8, 3.2; *P* = 0.42), language (97.2 ± 12.2 versus 97.9 ± 11.5, MD −0.7; 95 % CI −7.0, 5.6; *P* = 0.83) or motor (93.9 ± 10.8 versus 92.4 ± 9.7, MD 1.4; 95 % CI −4.0, 6.9; *P* = 0.61) development (Table 2). Adjustment for sex of the children did not change the outcome (data not shown). There were no differences in the percentage of children with any or moderate/severe developmental delay or in the parent-reported social-emotional behaviours and adaptive behaviour scores between the groups (Table 2).

**Table 2:** Developmental outcomes from the Bayley Scales of Infant and Toddler Development (Bayley-III)

Outcome	Iodine (*n* = 27)	Placebo (*n* = 26)	Treatment effect (95 % CI)	*P* value
Cognitive Standardised Score^a^	99.4 (12.2)	101.7 (8.2)	−2.3 (−7.8, 3.2)	0.42
Language Standardised Score^a^	97.2 (12.2)	97.9 (11.5)	−0.7 (−7.0, 5.6)	0.83
Motor Standardised Score^a^	93.9 (10.8)	92.4 (9.7)	1.4 (−4.0, 6.9)	0.61
Social-Emotional Standardised Score^a^	105.8 (15.9)	105.4 (16.2)	0.4 (−8.5, 9.3)	0.93
Adaptive Behaviour Standardised Score^a^	105.2 (15.2)	103.5 (14.9)	1.8 (−6.4, 10.0)	0.67
Cognitive score^b^ <85	1 (3.7)	0 (0.0)	N/A	>0.99
Cognitive score^b^ <70	1 (3.7)	0 (0.0)	N/A	>0.99
Language score^b^ <85	3 (11.1)	3 (11.5)	N/A	>0.99
Language score^b^ <70	0 (0.0)	0 (0.0)	N/A	N/A
Motor score^b^ <85	2 (7.4)	5 (19.2)	N/A	0.25
Motor score^b^ <70	1 (3.7)	0 (0.0)	N/A	>0.99

*CI* confidence interval, *N/A* not applicable

^a^The data are presented as mean (standard deviation), and the treatment effect is the difference in means

^b^The data are presented as number (percentage)

### Iodine status and thyroid function of mothers and infants

**Fig. 2:**
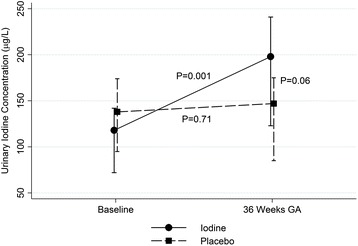
Median urinary iodine concentration of women. *GA* gestational age

The median (interquartile range) UICs of women at baseline (15 weeks’ gestation) and at 36 weeks’ gestation are shown in Fig. 2. The UIC increased from baseline to 36 weeks in the iodine group (median change (interquartile range) from baseline was 87 (−1 to 134) μg/l, *P* = 0.001) but not the placebo group (−2 (−76 to 37) μg/l, *P* = 0.71). The median (interquartile range) breast milk iodine concentration at 6 weeks after birth was 107 (79–147) μg/l overall, and there was no difference in breast milk concentration between the iodine and the placebo group (Table 3). Similarly there were no differences in cord blood fT3, fT4, TSH and Tg concentration between the groups (Table 3). Mean TSH of newborn or percentage of newborn with TSH of more than 5 mU/l from the routine newborn screen test also did not differ between the groups.

**Table 3:** Biomarkers of iodine status

	Iodine	Placebo	Effect (95 % CI)	*P* value
Cord blood	*n* = 19	*n* = 22		
Free triiodothyronine, pmol/l^a^	2.3 (0.4)	2.3 (0.6)	0.0 (−0.4, 0.3)	0.81
Free thyroxine, pmol/l^a^	14.4 (2.1)	13.8 (2.3)	0.6 (−0.9, 2.2)	0.40
Thyroid-stimulating hormone, mIU/l^b^	8.2 (5.9–13.5)	6.6 (4.5–9.6)	N/A	0.25
Thyroglobulin, μg/l^b^	73.0 (48.0–100.0)	64.0 (44.0–121.0)	N/A	0.66
	(n = 28)	(n = 29)		
Newborn TSH, mIU/l^a^	2.1 (1.0)	2.2 (1.2)	−0.1 (−0.7, 0.5)	0.79
Newborn TSH > 5^c^	0 (0.0)	0 (0.0)	N/A	N/A
	(n = 20)	(n = 25)		
Breast milk iodine at 6 weeks’ post-partum, μg/l^b^	106.0 (84.0–146.0)	124.0 (76.0–155.0)	N/A	0.74

*CI* confidence interval, *N/A* not applicable, *TSH* thyroid-stimulating hormone

^a^Data are presented as mean (standard deviation), and the treatment effect is the difference in means

^b^Data are presented as median (interquartile range)

^c^Data are presented as number (percentage)

### Pregnancy and other clinical outcomes

The mean birth weight, length, head circumference and gestational age at birth did not differ between the groups (Table 4). Adjustment for infant sex did not change the outcome (data not shown). The percentage of infants classified as low birth weight (<2500 g) or small for gestational age or large for gestational age, or with a neonatal complication or major congenital abnormality, did not differ between the treatment and placebo groups (Table 4). Other pregnancy outcomes, including rate of preterm birth, miscarriage, still birth and antenatal hospital admission, were also not different between the groups (Table 4).

**Table 4:** Pregnancy and neonatal outcomes

	Iodine	Placebo	Treatment effect (95 % CI)	*P* value
Pregnancy outcome	*n* = 29	*n* = 29		
Miscarriage^a^	1 (3.5)	0 (0.0)	N/A	>0.99
Stillbirth^a^	0 (0.0)	0 (0.0)	N/A	N/A
Caesarean section^a^	9 (32.1)	5 (17.2)	N/A	0.19
Post-term induction^a^	3 (10.7)	4 (13.8)	N/A	>0.99
Gestational diabetes^a^	1 (3.6)	2 (6.9)	N/A	>0.99
Pregnancy-induced hypertension^a^	0 (0.0)	0 (0.0)	N/A	N/A
Pre-eclampsia^a^	0 (0.0)	0 (0.0)	N/A	N/A
Antenatal hospital admission^a^	6 (22.2)	7 (24.1)	N/A	0.87
Neonatal outcome	n = 28	n = 29		
GA at birth, weeks^b^	39.3 (37.8 – 40.4)	39.7 (39.3 – 40.3)	N/A	0.23
Preterm birth, GA < 37 weeks^a^	5 (17.9)	4 (13.8)	N/A	0.73
Birth weight, g^c^	3325.4 (474.7)	3204.3 (689.4)	121.1 (−194.2, 436.3)	0.45
Birth length, cm^c^	49.4 (2.3)	48.7 (3.3)	0.7 (−0.9, 2.2)	0.37
Birth head circumference, cm^c^	34.6 (1.3)	33.9 (2.2)	0.7 (−0.3, 1.7)	0.15
Placental weight, g^c^	533.5 (136.2)	514.4 (129.5)	19.1 (−114.9, 153.2)	0.77
Low birth weight, <2500 g^a^	1 (3.6)	3 (10.3)	N/A	0.61
SGA^a^	3 (10.7)	2 (6.9)	N/A	0.67
LGA^a^	2 (7.1)	4 (13.8)	N/A	0.67
Major congenital abnormality^a^	0 (0.0)	0 (0.0)	N/A	N/A
Neonatal complication^a^	5 (17.9)	5 (17.2)	N/A	>0.99
Admission to NICU^a^	0 (0.0)	2 (6.9)	N/A	0.49
Neonatal death^a^	0 (0.0)	0 (0.0)	N/A	N/A

*CI* confidence interval, *N/A* not applicable, *GA* gestational age, *SGA* small for gestational age, *LGA* large for gestational age, *NICU* neonatal intensive care unit

^a^Data are presented as number (percentage)

^b^Data are presented as median (interquartile range)

^c^Data are presented as mean (standard deviation)

No women had medically diagnosed depression in pregnancy, and one woman in the iodine group had post-natal depression. There were no differences in the SF-36 or DASS outcomes or the frequency of sweating and palpitation, gastrointestinal side effects or any serious adverse events between the treatment groups (data not shown).

## Discussion

Our study is the first randomised, double-blind, placebo-controlled trial conducted in an industrialised country to assess the effect of routine iodine supplementation in pregnancy on childhood development. Although the unforeseeable early cessation of the trial resulted in a shorter duration of intervention and a significant reduction in the sample size, which may bias our results toward a null finding, we found no consistent trend of a higher or lower mean score in the neurodevelopmental outcomes between the iodine-supplemented and the placebo groups. Based on a recent national health survey of school-age children and non-pregnant adults, including childbearing aged women, Australia is no longer iodine-deficient after mandatory iodine fortification [20]. The question of whether iodine supplementation in pregnancy is required over and above the mandatory iodine fortification in Australia remains unanswered.

Despite the small sample size, our findings are consistent with the only two published RCTs conducted in regions of severe iodine deficiency more than three decades ago [21, 22], which showed no difference in IQ or cognitive development of children between the iodine-supplemented and the control groups in the absence of overt iodine deficiency (i.e., cretinism). Concerns that mild iodine deficiency may lead to cognitive impairment were based largely on two non-randomised intervention studies which showed that children whose mothers commenced iodine supplements in the first trimester had better neurodevelopment than children whose mothers took iodine supplements in the third trimester [23] or no supplements in pregnancy [4]. However, both studies have major methodological limitations and small sample sizes, with only 13 % of the original cohort selected for developmental assessment in one of the studies [23]. Thus, the results are likely to be subject to bias. Currently there is a lack of evidence from RCTs investigating the effect of iodine supplementation in pregnancy on childhood development in populations of mild to moderate iodine deficiency. Findings from cohort studies investigating the relationship between mild iodine deficiency in pregnancy, defined as maternal UIC of less than 150 μg/l, and neurodevelopmental outcomes of children are inconsistent. Whereas two cohort studies showed that mild to moderate maternal iodine deficiency in early pregnancy was associated with lower IQ [24] and reduced educational outcomes [25], other cohort studies showed no difference in the developmental outcomes of children between mothers who had mild to moderate iodine deficiency or iodine sufficiency [5, 26].

The mean cognitive and language scores of the children in our study are comparable to a large sample of children (the DOMInO study [27]) prior to mandatory iodine fortification of bread in Australia. The mean composite motor score of children in our study is approximately half a standard deviation below the population mean and is considerably lower than the children in the DOMInO study [27]. Our study sample is unlikely to be representative of the general population, and this may partly explain the lower motor score, although the effect of mandatory iodine fortification in Australia on child development is unknown. A recent larger Spanish cohort study (>1500 mother-and-child pairs) in regions of iodine sufficiency showed that maternal intake of multivitamin supplements containing at least 150 μg of iodine per day was associated with an increased risk of Bayley motor score of less than 85 in children at 1 year of age compared with iodine supplements containing less than 100 μg per day [5]. This suggests that potential adverse effects of iodine supplementation in pregnancy at the recommended dose of 150 μg/d in regions of iodine sufficiency like Australia post-mandatory iodine fortification cannot be excluded in the absence of quality RCTs.

We observed no differences in markers of thyroid function in cord blood or newborns between the groups, and this is consistent with findings from systematic reviews of RCTs [3, 28] in regions of mild to moderate iodine deficiency where the majority of the trials found no differences in thyroid hormone concentration between the iodine-supplemented and the control groups. This is in contrast to RCTs in the regions of severe iodine deficiency and suggests that pregnant women in regions of mild to moderate iodine deficiency are able to maintain adequate thyroid hormone production to meet increased requirements in pregnancy, and this may partly explain the lack of benefit of iodine supplementation in pregnancy on child development observed in our study.

## Conclusions

There are widespread recommendations for routine iodine supplementation in pregnancy, yet the efficacy and safety of routine iodine supplementation in pregnancy in a population with mild iodine deficiency or iodine sufficiency remain unclear. Although placebo-controlled randomised trials in such populations are viewed by some as unethical, conversely recommendations made in the absence of quality evidence also raise issues of ethical responsibility for clinicians and may result in lower compliance with such recommendations. A definitive RCT with an adequate sample size is warranted to provide the rigorous evidence necessary to inform clinical practice and public health policy in order to provide the best care for pregnant women and optimal growth and development of their children.

## Abbreviations

Bayley-III: Bayley Scales of Infant and Toddler Development, third edition
CI: Confidence interval
DASS: Depression Anxiety Stress Scale
fT3: Free triiodothyronine
fT4: Free thyroxine
IQ: Intelligence quotient
MD: Mean difference
NHMRC: National Health and Medical Research Council
RCT: Randomised controlled trial
SF-36: 36-Item Short Form Health Survey
Tg: Thyroglobulin
TSH: Thyroid-stimulating hormone
UIC: Urinary iodine concentration
WHO: World Health Organization
